# Mitochondrial DNA 5178 C/A polymorphism influences the effects of habitual smoking on the risk of dyslipidemia in middle-aged Japanese men

**DOI:** 10.1186/1476-511X-11-97

**Published:** 2012-08-02

**Authors:** Akatsuki Kokaze, Mamoru Ishikawa, Naomi Matsunaga, Kanae Karita, Masao Yoshida, Naoki Shimada, Tadahiro Ohtsu, Takako Shirasawa, Hirotaka Ochiai, Masao Satoh, Masayasu Hashimoto, Hiromi Hoshino, Yutaka Takashima

**Affiliations:** 1 Department of Public Health, Showa University School of Medicine, 1-5-8 Hatanodai, Shinagawa-ku, Tokyo, 142-8555, Japan; 2 Department of Public Health, Kyorin University School of Medicine, 6-20-2 Shinkawa, Mitaka-shi, Tokyo, 181-8611, Japan; 3 Mito Red Cross Hospital, 3-12-48 Sannomaru, Mito-shi, Ibaraki, 310-0011, Japan; 4 School of Medical Technology and Health, Faculty of Health and Medical Care, Saitama Medical University, 1397-1 Yamane, Hidaka-shi, Saitama, 350-1241, Japan

**Keywords:** Cigarette smoking, Hypo-HDL cholesterolemia, Hyper-LDL cholesterolemia, Hypertriglyceridemia, Mitochondrial DNA polymorphism, Personalized preventive medicine

## Abstract

**Background:**

Several genetic polymorphisms have been reported to modify the effects of smoking on serum lipid levels. The objective of this study was to investigate whether longevity-associated mitochondrial DNA 5178 (Mt5178) C/A polymorphism modifies the effects of habitual smoking on the risk of dyslipidemia in middle-aged Japanese subjects.

**Methods:**

A total of 394 male subjects (age, 53.9 ± 7.9 years; mean ± SD) were selected from among individuals visiting the hospital for regular medical check-ups. After Mt5178 C/A genotyping, a cross-sectional study assessing the joint effect of Mt5178 C/A polymorphism and cigarette smoking on the risk of hypo-high-density lipoprotein (HDL) cholesterolemia, hyper-low-density lipoprotein (LDL) cholesterolemia or hypertriglyceridemia was conducted.

**Results:**

For subjects with Mt5178C, the risk of hypo-HDL cholesterolemia increased with the number of cigarettes smoked daily (*P* for trend = 0.001). On the other hand, the association between Mt5178A genotype and the risk of hypo-HDL cholesterolemia did not appear to depend on the number of cigarettes smoked daily. For those with Mt5178A, the risk of hyper-LDL cholesterolemia or hypertriglyceridemia increased with cigarettes smoked daily (*P* for trend = 0.017 and *P* for trend = 0.002, respectively). However, the association between Mt5178C genotype and the risk of hyper-LDL cholesterolemia or hypertriglyceridemia did not depend on the number of cigarettes smoked daily.

**Conclusions:**

The present results suggest that Mt5178 C/A polymorphism modulates the effects of habitual smoking on the risk of dyslipidemia in middle-aged Japanese men.

## Introduction

Prevention of Coronary heart disease (CHD) is one of the major public health issues. The Global Burden of Disease Study showed that CHD is predicted as the leading cause of death not only in 1990 but also in 2020 [1]. Population-based follow-up studies showed that smoking is one of critical risk factors of CHD [2,3]. Cigarette smoking is assumed to be involved in vasomotor dysfunction, inflammation and modification of lipids, which are the components for atherogenesis [4]. Habitual smoking promotes detrimental changes in serum lipid profile. From analysis of published data, Craig et al. concluded that habitual smoking is associated with significantly lower serum high-density lipoprotein (HDL) cholesterol levels, and higher serum low-density lipoprotein (LDL) cholesterol levels and serum triglyceride levels [5]. However, from a cross-sectional and longitudinal analysis in a large Japanese cohort, Kuzuya et al. reported that serum LDL cholesterol levels are significantly lower in smokers than in non-smokers [6]. One of the reasons for these inconsistent results may be differences in genetic background.

Several genetic polymorphisms have been reported to influence the effect of smoking on lipid profiles [7-12]. One of these polymorphisms is mitochondrial DNA cytosine/adenine (Mt5178 C/A) polymorphism, which is also recognized as NADH dehydrogenase subunit-2 237 leucine/methionine (ND2-237 Leu/Met) polymorphism. The frequency of the Mt5178A genotype is significantly higher in Japanese centenarians than in the general population, thus Mt5178 C/A polymorphism is associated with longevity in Japanese [13]. Moreover, from clinical points of view, Japanese individuals with Mt5178A are more resistant to adult-onset lifestyle-related diseases, such as hypertension [14], diabetes [15], myocardial infarction [16,17] and cerebrovascular disorders [18], than those with Mt5178C. This longevity-associated Mt5178 C/A polymorphism subclinically influences the effects of habitual smoking on serum concentration of triglyceride [12]. Recently, this polymorphism is reported to modify the effects of alcohol consumption [19] or coffee intake [20] on the risk of dyslipidemia. These previous studies stimulated our clinical interests to investigate whether there is a combined effects of Mt5178 C/A polymorphism and habitual smoking on the risk of dyslipidemia or not.

This study aims to investigate whether longevity-associated Mt5178 C/A polymorphism modifies the effects of habitual smoking on the risk of hypo-HDL cholesterolemia, hyper-LDL cholesterolemia or hypertriglyceridemia in middle-aged Japanese male subjects.

## Subjects and methods

### Subjects

Participants were recruited from among individuals visiting the Mito Red Cross Hospital for regular medical check-ups between August 1999 and August 2000. This study was conducted in accordance with the Declaration of Helsinki and was approved by the Ethics Committee of the Kyorin University School of Medicine. Written informed consent was obtained from 602 volunteers before participation. Because the number of women was insufficient for classification into groups based on Mt5178 C/A genotype and smoking status, women were excluded. Patients taking antihyperlipidemic medication were excluded, and diabetic patients were also excluded because of the higher prevalence of dyslipidemia in diabetic patients when compared to non-diabetic patients [21]. Thus, 406 men were enrolled in the study. Twelve individuals with unclear data were also excluded. Therefore, subjects comprised 394 Japanese men (age, 53.9 ± 7.9 years; mean ± SD).

### Clinical characteristics of subjects

Determination of blood chemical and physical data was conducted as described previously [22]. According to the Japan Atherosclerosis Society guidelines for prevention of atherosclerotic cardiovascular diseases [23], hyper-LDL cholesterolemia as serum LDL cholesterol ≥ 140 mg/dl, hypo-HDL cholesterolemia as serum HDL cholesterol < 40 mg/dl and hypertriglyceridemia was defined as serum triglyceride ≥ 150 mg/dl. Body mass index (BMI) was defined as the ratio of subject weight (kg) to the square of subject height (m). LDL cholesterol levels were calculated by means of Friedewald’s formula [24]. A survey of habitual smoking, alcohol consumption and coffee consumption was performed by means of questionnaire. Smoking status was classified based on number of cigarettes smoked per day (never- or ex-smokers; 1–20 cigarettes smoked per day; and >20 cigarettes smoked per day). Alcohol consumption was classified based on drinking frequency (daily drinkers; occasional drinkers, which include those who drink several times per week or per month; and non- or ex-drinkers). Coffee consumption was classified based on number of cups of coffee per day (≤1 cup per day; 2–3 cups per day; and ≥4 cups per day). For use of antihypertensive medication, subjects were classified as taking no drug treatment or taking medicine.

### Genotyping

DNA was extracted from white blood cells using the DNA Extractor WB kit (Wako Pure Chemical Industries, Japan). The Mt5178 C/A polymorphism was detected by polymerase chain reaction (PCR) and digestion with *Alu*I restriction enzyme. The sequence of primers was: forward 5’-CTTAGCATACTCCTCAATTACCC-3’, reverse 5’-GTGAATTCTTCGATAATGGCCCA-3’. The PCR was performed with 50 ng genomic DNA in buffer containing 0.2 μmol/l of each primer, 1.25 mmol/l dNTPs, 1.5 mmol/l MgCl_2_, and 1 U of Taq DNA polymerase. After an initial denaturation at 94°C for 5 min, PCR was conducted through 40 cycles in the following steps: denaturation at 94°C for 30 s, annealing at 60°C for 60 s and polymerase extension at 72°C for 90 s. After cycling, a final extension at 72°C for 10 min was performed. The PCR products were digested with *Alu*I restriction enzyme (Nippon Gene, Japan) at 37°C overnight, and electrophoresed in 1.5% agarose gels stained with ethidium bromide for visualization under ultraviolet light. The absence of an *Alu*I site was designated as Mt5178A, and the presence of this restriction site was designated as Mt5178C.

### Statistical analyses

Statistical analyses were performed using SAS statistical software, version 9.2 for Windows. Multiple logistic regression analysis was used to calculate odds ratios (OR) for hypo-HDL cholesterolemia, hyper-LDL cholesterolemia or hypertriglyceridemia. For multiple logistic regression analysis and analysis of covariance, habitual alcohol consumption (non- or ex- drinkers = 0, occasional- drinkers = 1, daily- drinkers = 2), coffee consumption (≤1 cup per day = 1, 2–3 cups per day = 2, ≥4 cups per day = 3) and antihypetensive medication use (no use of antihypetensive = 0, use of antihypetensive = 1) were numerically coded. Differences with P values of less than 0.05 were considered to be statistically significant.

## Results

No significant differences in biophysical or biochemical characteristics were observed between the Mt5178C and Mt5178A genotypes (Table 1). No significant differences in smoking status were observed between the Mt5178 C/A genotypes. From viewpoints of dyslipidemia, no significant differences in the frequency of hypo-HDL cholesterolemia, that of hyper-LDL cholesterolemia or that of hypertriglyceridemia were observed between men with Mt5178C and those with Mt5178A.

**Table 1:** Clinical characteristics of study subjects by Mt5178 C/A genotype

	**Mt5178C**	**Mt5178A**	***P*value**
	**N = 239**	**N = 155**	
Age (y)	54.3 ± 7.8	53.2 ± 7.8	0.171
Body Mass Index (kg/m^2^)	23.3 ± 2.8	23.5 ± 2.6	0.461
Serum total cholesterol (mg/dl)	203.5 ± 34.3	202.1 ± 31.8	0.672
Serum HDL cholesterol (mg/dl)	54.6 ± 13.6	56.3 ± 16.2	0.269
Serum LDL cholesterol (mg/dl)	121.6 ± 34.8	117.9 ± 30.5	0.281
Serum Triglyceride (mg/dl)	136.7 ± 91.1	139.5 ± 90.8	0.766
Systolic blood pressure (mmHg)	125.8 ± 15.9	125.7 ± 14.1	0.940
Diastolic blood pressure (mmHg)	74.0 ± 10.7	73.8 ± 9.1	0.823
Fasting plasma glucose (mg/dl)	97.2 ± 9.4	97.6 ± 9.7	0.672
Smoking status (never- or ex-/ 1–20 cigarettes per day/ >20 cigarettes per day) (%)	59.4/29.7/10.9	59.4/25.8/14.8	0.429
Alcohol consumption (daily/occasionally/non- or ex-) (%)	46.4/35.2/18.4	47.7/38.7/13.6	0.426
Coffee consumption (≤ 1 cup per day/2-3 cups per day/≥ 4 cups per day) (%)	61.1/29.7/9.2	55.5/32.9/11.6	0.510
Antihypertensive medication use (%)	18.8	12.9	0.122
Hypo-HDL cholesterolemia (%)	11.7	12.3	0.871
Hyper-LDL cholesterolemia (%)	26.4	25.8	0.903
Hypertriglyceridemia (%)	29.7	29.7	0.995

HDL; high-density lipoprotein. LDL; low-density lipoprotein. Age, body mass index, serum HDL cholesterol levels, serum LDL cholesterol levels, serum triglyceride levels, systolic blood pressure, diastolic blood pressure, fasting plasma glucose levels are given as means ± S.D. For smoking status, coffee consumption, alcohol consumption, antihypertensive medication use, hypo-HDL cholesterolemia, hyper-LDL cholesterolemia and hypertriglyceridemia, *P* values were calculated by chi-squared test. All *P* values depict significance of differences between Mt5178C and Mt5178A.

For Mt5178C genotypic men, after adjustment for age and BMI or for age, BMI, alcohol consumption, coffee consumption and use of antihypertensive medication, number of cigarettes smoked per day was negatively and significantly associated with serum HDL cholesterol levels (adjusted *P* for trend = 0.008 and adjusted *P* for trend = 0.037, respectively) (Table 2). For Mt5178A genotypic men, this negative association between number of daily cigarettes smoked and serum HDL levels did not reach to significant level. However, for Mt5178A genotypic men, number of cigarettes smoked per day was positively and significantly associated with serum triglyceride levels (*P* for trend < 0.001). After adjustment, this positive association between number of cigarette consumed per day and serum triglyceride levels remained significant level. Moreover, after adjustment or not, serum triglyceride levels were significantly higher in those who consumed >20 cigarettes per day than in never- or ex-smokers or in subjects who consumed 1–20 cigarettes per day (*P* < 0.001 and *P* < 0.001, respectively).

**Table 2:** Serum lipid levels among three smoking status by Mt5178 C/A genotypes

	**Smoking status**			***P*for trend**
	**Never- or ex-smokers**	**1-20 cigarettes smoked per day**	**>20 cigarettes smoked per day**	
**Mt5178C**	N = 142	N = 70	N = 27	
HDL cholesterol	55.9 ± 1.1	52.6 ± 1.6	52.6 ± 2.6	0.089
HDL cholesterol †	56.5 ± 1.1	52.1 ± 1.5	51.0 ± 2.5	0.008
HDL cholesterol ‡	55.6 ± 1.6	52.2 ± 1.8	51.1 ± 2.7	0.037
LDL cholesterol ‡	122.2 ± 2.9	116.8 ± 4.1	130.7 ± 6.7	0.672
LDL cholesterol †	121.8 ± 2.9	117.2 ± 4.2	131.9 ± 6.8	0.514
LDL cholesterol ‡	126.7 ± 4.5	119.7 ± 5.0	134.6 ± 7.4	0.795
Triglyceride	134.7 ± 7.7	139.6 ± 10.9	139.5 ± 17.6	0.717
Triglyceride†	133.3 ± 7.5	141.0 ± 10.7	143.2 ± 17.3	0.502
Triglyceride‡	125.6 ± 11.6	135.8 ± 13.0	138.5 ± 19.3	0.394
**Mt5178A**	N = 92	N = 40	N = 23	
HDL cholesterol	57.5 ± 1.7	56.2 ± 2.6	51.5 ± 3.4	0.136
HDL cholesterol †	58.0 ± 1.6	54.1 ± 2.5	53.0 ± 3.2	0.100
HDL cholesterol ‡	56.3 ± 2.6	52.1 ± 2.9	50.7 ± 3.8	0.071
LDL cholesterol	115.8 ± 3.2	119.6 ± 4.8	123.2 ± 6.4	0.266
LDL cholesterol †	115.1 ± 3.2	122.1 ± 4.9	121.8 ± 6.3	0.221
LDL cholesterol ‡	118.3 ± 5.1	122.6 ± 5.8	123.3 ± 7.6	0.392
Triglyceride	120.4 ± 8.6	130.9 ± 13.1	230.7 ± 17.3*^,^**	<0.001
Triglyceride†	119.4 ± 8.5	136.8 ± 13.1	224.6 ± 17.0*^,^**	<0.001
Triglyceride‡	114.5 ± 13.6	137.4 ± 15.4	223.5 ± 20.4*^,^**	<0.001

HDL; high-density lipoprotein. LDL; low-density lipoprotein. †HDL cholesterol levels, †LDL cholesterol levels and †triglyceride levels are given as least-square mean ± S.E. adjusted for age, body mass index; ‡HDL cholesterol levels , ‡LDL cholesterol levels and ‡triglyceride levels are given as least-square mean ± S.E. adjusted for age, body mass index, alcohol consumption, coffee consumption and antihypertensive medication use. The Bonferroni correction for multiple comparison was applied. *P < 0.001 vs. never- or ex-smokers, **P < 0.001 vs. current smokers who smoked 1–20 cigarettes per day.

For subjects with Mt5178C, the risk of hypo-HDL cholesterolemia may depend on number of daily cigarettes smoked (*P* for trend = 0.001) (Table 3). After adjustment, positive association between increasing number of daily cigarettes smoked and the risk of hypo-HDL cholesterolemia remained significantly. The crude OR for hypo-HDL cholesterolemia was significantly higher in subjects who consumed 1–20 cigarettes per day or in those who consumed >20 cigarettes per day than in never- or ex-smokers (OR = 3.058, 95% confidence interval (CI): 1.221–7.654, *P* = 0.017 and OR = 5.172, 95% CI: 1.732–15.44, *P* = 0.003, respectively). After adjustment for age and BMI or for age, BMI, habitual alcohol consumption, coffee consumption and use of antihypertensive medication, a significant OR remained. On the other hand, the association between Mt5178A genotype and the risk of hypo-HDL cholesterolemia does not appear to depend on number of daily cigarettes smoked.

**Table 3:** Odds ratios (ORs) and 95% confidence intervals (CIs) for hypo-HDL cholesterolemia by Mt5178 C/A genotype and smoking status

**Genotype and number of cigarette smoked daily**	**Frequency**		**OR (95% CI)**	**Adjusted OR† (95% CI)**	**Adjusted OR‡ (95% CI)**
	**Normal HDLcholesterol (HDL cholesterol ≥ 40 mg/dl)**	**Hypo-HDLcholesterolemia (HDL cholesterol < 40 mg/dl)**			
**Mt5178C**					
0 (never- or ex-smoker)	133 (93.7)	9 (6.3)	1 (reference)	1 (reference)	1 (reference)
1-20	58 (82.9)	12 (17.1)	3.058 (1.221-7.654)*	3.317 (1.303-8.446)*	2.771 (1.039-7.388)*
>20	20 (74.1)	7 (25.9)	5.172 (1.732-15.44)**	8.048 (2.244-28.87)**	8.339 (1.993-34.89)**
			*P* for trend = 0.001	*P* for trend < 0.001	*P* for trend = 0.002
**Mt5178A**					
0 (never- or ex-smoker)	82 (89.1)	10 (10.9)	1 (reference)	1 (reference)	1 (reference)
1-20	34 (85.0)	6 (15.0)	1.447 (0.488-4.297)	2.143 (0.664-6.918)	1.703 (0.481-6.027)
>20	20 (87.0)	3 (13.0)	1.230 (0.310-4.888)	1.158 (0.281-4.781)	1.264 (0.289-5.533)
			*P* for trend = 0.629	*P* for trend = 0.563	*P* for trend = 0.577

†OR adjusted for age and body mass index.

‡OR adjusted for age, body mass index, habitual alcohol consumption, coffee consumption and antihypertensive medication.

*P < 0.05, **P < 0.005.

For subjects with Mt5178A, the risk of hyper-LDL cholesterolemia may depend on number of daily cigarettes smoked (*P* for trend = 0.017) (Table 4). After adjustment, increasing daily cigarettes smoked of the risk of hyper-LDL cholesterolemia remained significantl level. The crude OR for hyper-LDL cholesterolemia was significantly higher in subjects who consumed 1–20 cigarettes per day or in those who consumed >20 cigarettes per day than in never- or ex-smokers (OR = 2.376, 95% CI: 1.029–5.482, *P* = 0.043 and OR = 2.836, 95% CI: 1.055–7.626, *P* = 0.039, respectively). After adjustment, a OR remained significant. For them, the risk of hypertriglyceridemia may also depend on number of daily cigarettes smoked (*P* for trend = 0.002) (Table 5). After adjustment, increasing daily cigarettes smoked of the risk of hypertriglyceridemia remained significant. The crude OR for hypertriglyceridemia was significantly higher in subjects who consumed >20 cigarettes per day than in never- or ex-smokers (OR = 5.966, 95% CI: 2.233–15.90, *P* < 0.001). After adjustment, a significant OR remains. On the other hand, the association between Mt5178C genotype and the risk of hyper-LDL cholesterolemia or that of hypertriglyceridemia does not seem to depend on number of daily cigarettes smoked (Tables 4, 5).

**Table 4:** Odds ratios (ORs) and 95% confidence intervals (CIs) for hyper-LDL cholesterolemia by Mt5178 C/A genotype and smoking status

**Genotype and number of cigarette smoked daily**	**Frequency**		**OR (95% CI)**	**Adjusted OR† (95% CI)**	**Adjusted OR‡ (95% CI)**
	**Normal LDLcholesterol (LDL cholesterol < 140 mg/dl)**	**Hyper-LDLcholesterolemia (LDL cholesterol ≥ 140 mg/dl)**			
**Mt5178C**					
0 (never- or ex-smoker)	107 (75.4)	35 (24.6)	1 (reference)	1 (reference)	1 (reference)
1-20	53 (75.7)	17 (24.3)	0.981 (0.504-1.910)	0.972 (0.492-1.923)	0.944 (0.460-1.940)
>20	16 (59.3)	11 (40.7)	2.102 (0.892-4.953)	2.181 (0.889-5.348)	2.506 (0.973-6.457)
			*P* for trend = 0.181	*P* for trend = 0.160	*P* for trend = 0.164
**Mt5178A**					
0 (never- or ex-smoker)	75 (81.5)	17 (18.5)	1 (reference)	1 (reference)	1 (reference)
1-20	26 (65.0)	14 (35.0)	2.376 (1.029-5.482)*	2.993 (1.222-7.330)*	2.790 (1.080-7.206)*
>20	14 (60.9)	9 (39.1)	2.836 (1.055-7.626)*	2.982 (1.080-8.232)*	2.908 (1.012-8.351)*
			*P* for trend = 0.017	*P* for trend = 0.014	*P* for trend = 0.035

†OR adjusted for age and body mass index.

‡OR adjusted for age, body mass index, habitual alcohol consumption, coffee consumption and antihypertensive medication.

*P < 0.05, **P < 0.005.

**Table 5:** Odds ratios (ORs) and 95% confidence intervals (CIs) for hypertriglyceridemia by Mt5178 C/A genotype and smoking status

**Genotype and number of cigarette smoked daily**	**Frequency**		**OR (95% CI)**	**Adjusted OR† (95% CI)**	**Adjusted OR‡ (95% CI)**
	**Normal triglyceride level(Triglyceride < 150 mg/dl)**	**Hypertriglyceridemia(Triglyceride ≥ 150 mg/dl)**			
**Mt5178C**					
0 (never- or ex-smoker)	102 (71.8)	40 (28.2)	1 (reference)	1 (reference)	1 (reference)
1-20	48 (68.6)	22 (31.4)	1.169 (0.627-2.180)	1.226 (0.640-2.350)	1.355 (0.681-2.697)
>20	18 (66.7)	9 (33.3)	1.275 (0.529-3.073)	1.429 (0.563-3.631)	1.517 (0.575-3.998)
			*P* for trend = 0.517	*P* for trend = 0.366	*P* for trend = 0.282
**Mt5178A**					
0 (never- or ex-smoker)	70 (76.1)	22 (23.9)	1 (reference)	1 (reference)	1 (reference)
1-20	31 (77.5)	9 (22.5)	0.924 (0.382-2.234)	1.261 (0.485-3.284)	1.588 (0.537-4.697)
>20	8 (34.8)	15 (65.2)	5.966 (2.233-15.90)**	5.629 (2.071-15.30)**	6.397 (2.177-18.79)**
			*P* for trend = 0.002	*P* for trend = 0.002	*P* for trend < 0.001

†OR adjusted for age and body mass index.

‡OR adjusted for age, body mass index, habitual alcohol consumption, coffee consumption and antihypertensive medication.

*P < 0.05, **P < 0.005.

## Discussion

In the present study, we observed that longevity-associated Mt5178 C/A polymorphism may influence the effects of habitual smoking on increasing the risk of hypo-HDL cholesterolemia, that of hyper-LDL cholesterolemia or that of hypertriglyceridemia in middle-aged Japanese men. For men with Mt5178C, who are more susceptible to atherosclerotic diseases than those with Mt5178A, habitual smoking may increase the risk of hypo-HDL cholesterolemia. On the other hand, for men with Mt5178A, cigarette smoking may increase the risk of hyper-LDL cholesterolemia or that of hypertriglyceridemia. These findings are new gene-environment interactions on the risk of dyslipidemia and may contribute to establishment of individualized prevention for dyslipidemia.

Meta-analysis conducted by Craig et al. showed that smokers have significantly lower serum HDL cholesterol levels, and higher serum LDL cholesterol levels and serum triglyceride levels [5]. However, the large-scale cross-sectional and longitudinal analysis showed that serum LDL cholesterol levels in smokers are significantly lower than those in non-smokers [6]. Genetic epidemiological approaches may be useful for elucidating this inconsistency in the effects of habitual smoking on serum lipid profiles. In addition to our results, several joint effects of genetic factor and cigarette smoking on HDL cholesterol levels [7-10], LDL cholesterol levels [9] or triglyceride levels [11,12] have been already published. The polymorphism in *PNPN11* gene, encoding Src homology 2 domain-containing protein tyrosine phosphatase-2, modulates the effect of cigarette smoking on serum HDL cholesterol levels and serum LDL cholesterol levels [9]. Among the *PTPN11* (rs2301756) AA genotype, asignificant increase in HDL cholesterol levels and a significant decrease in LDL cholesterol levels in current smokers compared to non-smokers [9]. Moreover, more than twenty genetic variants are reported to be associated with modulated lipid levels, and some of them are also associated with the risk of CHD [25]. Therefore, these reports and our results inspire to investigate gene-gene or gene-gene-environment interactions, for example lipoprotein lipase gene-apolipoprotein C-III gene-smoking interaction [7], on serum lipids levels, the risk of dyslipidemia or that of CHD.

For men with Mt5178C, habitual smoking may significantly decrease the serum HDL cholesterol levels and significantly increase the risk of hypo-HDL cholesterolemia. For those with Mt5178A, habitual smoking may significantly increase both the serum triglyceride levels and the risk of hypertriglyceridemia. In these observations, joint effects of Mt5178 C/A polymorphism and habitual smoking on the risk of dyslipidemia are consistent with those on serum lipid profiles. However, for men with Mt5178A, cigarette smoking may not significantly increase the serum LDL cholesterol levels but may significantly increase the risk of hyper-LDL cholesterolemia. Originally, diagnostic criteria for dislipidemia, namely hyper-LDL cholesterolemia, hypo-HDL cholesterolemia and hypertriglyceridemia, was proposed for the prevention of atherosclerotic diseases, especially CHD [23]. From viewpoints of preventive medicine, detection of the genetic risk for dyslipidemia is more clinically important than that of genetic factors influencing serum lipid levels. Therefore, determination of the Mt5178 C/A genotype is thought to be practical.

 Our previous investigations revealed that daily consumption of ≥ 1 cup of coffee may increase the risk of hyper-LDL cholesterolemia for Mt5178A genotypic men [20]. Clinically interestingly, daily alcohol consumption may reduce the risk of hyper-LDL cholesterolemia for men with Mt5178C [19] and decrease serum triglyceride levels for those with Mt5178A [12]. Makita et al. reported that yearly changes in serum LDL cholesterol levels were significantly lower in non-daily drinkers with Mt5178A than in those with Mt5178C [26]. Therefore, the determination of Mt5178 C/A genotypes seems productive of establishing individualized prevention of dyslipidemia. Moreover, based on the information of Mt5178 C/A polymorphism, personalized behavior modifications in lifestyle, namely smoking cessation, possibly contribute to establishing personalized prevention for hypo-HDL cholesterolemia among Mt5178C genotypic men, for hyper-LDL cholesterolemia or that of hypertriglyceridemia among Mt5178A genotypic men and then for subsequent life-threatening atherosclerotic diseases among the both genotypic men. Actually, the meta-analysis showed that smoking cessation increases HDL cholesterol level [27]. However, Yoon et al. disclosed that smoking cessation-associated harmful changes in the risk factors for CHD were predominantly secondary to weight gain, and advocated attentive weight control in smoking quitters in order to reduce the risk of CHD [28].

The mechanism underlying the combined effects of Mt5178 C/A polymorphism and habitual smoking on the risk of dyslipidemia has not been elucidated. Individuals with the Mt5178A genotype are more resistant to atherosclerotic diseases than those with the Mt5178C genotype [16-18]. These antiatherogenic advantages are presumed to be brought from the biophysical and biochemical properties of ND2-237Met, which is genetically coded by Mt5178A. NADH dehydrogenase, namely respiratory chain complex I, is considered as the major physiological and pathological site of reactive oxygen species (ROS) generation in mitochondria, and as a target of attack by ROS [29]. Mitochondrial DNA is also vulnerable to damage by ROS [29]. Increased mitochondrial ROS production, mitochondrial dysfunction and mitochondrial DNA mutation are associated with atherosclerotic diseases [29]. Mouse mitochondrial DNA 4738 C/A (Mt4738 C/A) polymorphism also results in a leucine to methionine substitution in NADH dehydrogense subunit 2. Gusdon et al. showed that NADH dehydrogense ROS production is significantly lower in Mt4738A genotypic mice than in Mt4738C genotypic mice [30]. Extrapolation from experimental mice to human may assume that ND2-237Met suppresses ROS production. Moreover, deliberating on that methionine residues play an antioxidant role in scavenging ROS [31], ND2-237Met may also protect NADH dehydrogenase itself and mitochondrial DNA from ROS. Cigarette smoke contains high concentrations of ROS, and is supposed to activate endogenous sources of ROS [4]. There may be biophysical and biochemical differences in the protection against cigarette smoking-induced ROS or the reduction of cigarette smoking-induced ROS generation between ND2-237Leu and ND2-237Met. These deduced differences are conjectured to bring that Mt5178 C/A polymorphism modifies the effects of cigarette smoking on the risk of hypo-HDL cholesteloremia, hyper-LDL cholesterolemia or hypertriglyceridemia. We cannot prepare either satisfactory elucidation to the discrepancy between combined effects of Mt5178A genotype and cigarette smoking on serum LDL cholesterol levels and those on the risk of hyper-LDL cholesterolemia. Considering that mitochondrial DNA is maternally inherited, a possibility is speculated that there is a genetic linkage between Mt5178A genotype and the other mitochondrial DNA genotype affecting the risk of hyper-LDL cholesterolemia. Anyway, in order to reveal the mechanisms responsible for interaction between ND2-237 Leu/Met genotypes and habitual smoking on the risk of dyslipidemia, further molecular biochemical and pharmacological inquiries are required.

In interpreting our results, it is necessary to contemplate the validity of the study design. The lifestyle habits, especially diet, probably vary between smokers and non-smokers. Systematic reviews manifest the effects of diets on serum lipids levels [32,33]. Hence, diet survey is required. The study sample size was not sufficient to test the gene-environment interaction. Chi-squared test does not show a significant statistical difference in the frequency of Mt5178A between in this study and in the large-scale molecular epidemiological survey [34], thus suggesting that there is no critical genetic bias in the subjects in this study. However, we cannot deny selection bias due to selection of subjects from those visiting the hospital for regular medical check-ups. Based on the cross-sectional as well as longitudinal study, Kuzuya et al. speculated that the effects of habitual smoking on serum lipid levels are influenced by age [6]. Additionally, although cross-sectional studies can contribute suggestive causal links, they cannot establish a valid causality. Therefore, in order to conquer these limitations denoted above, a large-scale population-based follow-up study is necessary.

In conclusion, longevity-associated Mt5178 C/A polymorphism may modulate the effects of habitual smoking on the risk of dyslipidemia in middle-aged Japanese men. For Mt5178A genotypic men, cigarette smoking may increase the risk of hyper-LDL cholesterolemia or that of hypertriglyceridemia and may squander their genetic resistance against atherosclerosis. On the other hand, for Mt5178C genotypic men, habitual smoking may increase the risk of hypo-HDL cholesterolemia and may coerce them more susceptible to subsequent atherosclerotic diseases. Genetic information of Mt5178 C/A polymorphism may propose the personalized behavioral modification for controlling of dyslipidemia, thereby clinically reducing the incidence of CHD. Moreover, this new gene-environmental interaction may contribute to elucidation of molecular pathophysiology of smoking- or mitochondria-related atherogenesis.

## Competing interests

The authors declare that they have no competing interests.

## Authors’ contributions

AK designed the study, carried out the epidemiological survey, carried out the genotyping, analyzed the data, and drafted the manuscript; MI collected the samples; NM helped to carry out the genotyping; KK and MY carried out the epidemiological survey; NS, TO, TS, HO, MS, MH, and HH assisted in data analysis and helped with interpreting the results; YT designed the study and carried out the epidemiological survey. All authors have read and approved the final manuscript.
